# Supplementary material for: Spinal Deformities in Wild Reptiles: A Systematic Review and Meta-Analysis
Source: Biology (Basel). 2025 Aug 24;14(9):1119. doi: 10.3390/biology14091119 (PMC12466963; doi:10.3390/biology14091119)
Supplement: Supplementary file 1 [file biology-14-01119-s001.zip › biology-3814211-supplementary.pdf]

Supporting Information for: Reptilian hunchbacks: a review and meta-analysis of spinal deformities in wild reptiles.

Gergely Horváth[1,2]
*email: gergely.horvath@ttk.elte.hu
[1]Department of Systematic Zoology and Ecology, Institute of Biology, ELTE Eötvös Loránd University, Pázmány Péter sétány 1/c, 1117 Budapest, Hungary
[2]HUN-REN–ELTE–MTM Integrative Ecology Research Group, Pázmány Péter sétány 1/c, 1117 Budapest, Hungary

Table of contents

Supplementary Table S1. PRISMA 2020 Checklist. 2
Supplementary Figure S1. PRISMA diagram summarizing the literature search. 6
Supplementary Figure S2. Graphical summary of phylogenetic distribution of 35 species included in the meta-analyses. 7
Supplementary Figure. S3. Funnel plot and relationships between effect sizes and publication year. 8
Supplementary Table S2. Occurrence and prevalence of spinal deformations in the currently known families of reptiles. 9

Supplementary Table S1. PRISMA 2020 Checklist.

Section and Topic	Item #	Checklist item	Location where item is reported
**TITLE**			
Title	1	Identify the report as a systematic review.	L 1-2
**ABSTRACT**			
Abstract	2	See the PRISMA 2020 for Abstracts checklist.	L 31-47
**INTRODUCTION**			
Rationale	3	Describe the rationale for the review in the context of existing knowledge.	Section 1
Objectives	4	Provide an explicit statement of the objective(s) or question(s) the review addresses.	L 241-249
**METHODS**			
Eligibility criteria	5	Specify the inclusion and exclusion criteria for the review and how studies were grouped for the syntheses.	Section 2.1.
Information sources	6	Specify all databases, registers, websites, organisations, reference lists and other sources searched or consulted to identify studies. Specify the date when each source was last searched or consulted.	L 255-283
Search strategy	7	Present the full search strategies for all databases, registers and websites, including any filters and limits used.	L 252-270
Selection process	8	Specify the methods used to decide whether a study met the inclusion criteria of the review, including how many reviewers screened each record and each report retrieved, whether they worked independently, and if applicable, details of automation tools used in the process.	L 286-291
Data collection process	9	Specify the methods used to collect data from reports, including how many reviewers collected data from each report, whether they worked independently, any processes for obtaining or confirming data from study investigators, and if applicable, details of automation tools used in the process.	Section 2.2.
Data items	10a	List and define all outcomes for which data were sought. Specify whether all results that were compatible with each outcome domain in each study were sought (e.g. for all measures, time points, analyses), and if not, the methods used to decide which results to collect.	Section 2.2.
	10b	List and define all other variables for which data were sought (e.g. participant and intervention characteristics, funding sources). Describe any assumptions made about any missing or unclear information.	Section 2.2.
Study risk of	11	Specify the methods used to assess risk of bias in the included studies, including details of the tool(s) used, how	Section 2.2.

Section and Topic	Item #	Checklist item	Location where item is reported
bias assessment		many reviewers assessed each study and whether they worked independently, and if applicable, details of automation tools used in the process.	
Effect measures	12	Specify for each outcome the effect measure(s) (e.g. risk ratio, mean difference) used in the synthesis or presentation of results.	Not applicable
Synthesis methods	13a	Describe the processes used to decide which studies were eligible for each synthesis (e.g. tabulating the study intervention characteristics and comparing against the planned groups for each synthesis (item #5)).	Section 2.3.
	13b	Describe any methods required to prepare the data for presentation or synthesis, such as handling of missing summary statistics, or data conversions.	Section 2.3.
	13c	Describe any methods used to tabulate or visually display results of individual studies and syntheses.	Section 2.3.
	13d	Describe any methods used to synthesize results and provide a rationale for the choice(s). If meta-analysis was performed, describe the model(s), method(s) to identify the presence and extent of statistical heterogeneity, and software package(s) used.	Section 2.3.
	13e	Describe any methods used to explore possible causes of heterogeneity among study results (e.g. subgroup analysis, meta-regression).	Section 2.3.
	13f	Describe any sensitivity analyses conducted to assess robustness of the synthesized results.	Section 2.3.
Reporting bias assessment	14	Describe any methods used to assess risk of bias due to missing results in a synthesis (arising from reporting biases).	Section 2.3.
Certainty assessment	15	Describe any methods used to assess certainty (or confidence) in the body of evidence for an outcome.	Section 2.3.
**RESULTS**			
Study selection	16a	Describe the results of the search and selection process, from the number of records identified in the search to the number of studies included in the review, ideally using a flow diagram.	Section 3.1.; Supplementary Fig. S1
	16b	Cite studies that might appear to meet the inclusion criteria, but which were excluded, and explain why they were excluded.	Not applicable
Study characteristics	17	Cite each included study and present its characteristics.	Supplementary Table S2
Risk of bias in	18	Present assessments of risk of bias for each included study.	Supplementary

Section and Topic	Item #	Checklist item	Location where item is reported
studies			Table S2
Results of individual studies	19	For all outcomes, present, for each study: (a) summary statistics for each group (where appropriate) and (b) an effect estimate and its precision (e.g. confidence/credible interval), ideally using structured tables or plots.	Sections 3.1.-3.2.
Results of syntheses	20a	For each synthesis, briefly summarise the characteristics and risk of bias among contributing studies.	Sections 3.1.-3.2.
	20b	Present results of all statistical syntheses conducted. If meta-analysis was done, present for each the summary estimate and its precision (e.g. confidence/credible interval) and measures of statistical heterogeneity. If comparing groups, describe the direction of the effect.	Sections 3.1.-3.2.
	20c	Present results of all investigations of possible causes of heterogeneity among study results.	Sections 3.1.-3.2.
	20d	Present results of all sensitivity analyses conducted to assess the robustness of the synthesized results.	Not applicable
Reporting biases	21	Present assessments of risk of bias due to missing results (arising from reporting biases) for each synthesis assessed.	Sections 3.1.-3.2.
Certainty of evidence	22	Present assessments of certainty (or confidence) in the body of evidence for each outcome assessed.	Sections 3.1.-3.2.
**DISCUSSION**			
Discussion	23a	Provide a general interpretation of the results in the context of other evidence.	Sections 4.1.-4.4.
	23b	Discuss any limitations of the evidence included in the review.	Sections 4.1.-4.4.
	23c	Discuss any limitations of the review processes used.	Sections 4.1.-4.4.
	23d	Discuss implications of the results for practice, policy, and future research.	Section 5
**OTHER INFORMATION**			
Registration and protocol	24a	Provide registration information for the review, including register name and registration number, or state that the review was not registered.	L 283
	24b	Indicate where the review protocol can be accessed, or state that a protocol was not prepared.	L 854-859

Section and Topic	Item #	Checklist item	Location where item is reported
	24c	Describe and explain any amendments to information provided at registration or in the protocol.	Not applicable
Support	25	Describe sources of financial or non-financial support for the review, and the role of the funders or sponsors in the review.	L 862-863
Competing interests	26	Declare any competing interests of review authors.	L 876
Availability of data, code and other materials	27	Report which of the following are publicly available and where they can be found: template data collection forms; data extracted from included studies; data used for all analyses; analytic code; any other materials used in the review.	L 872-873

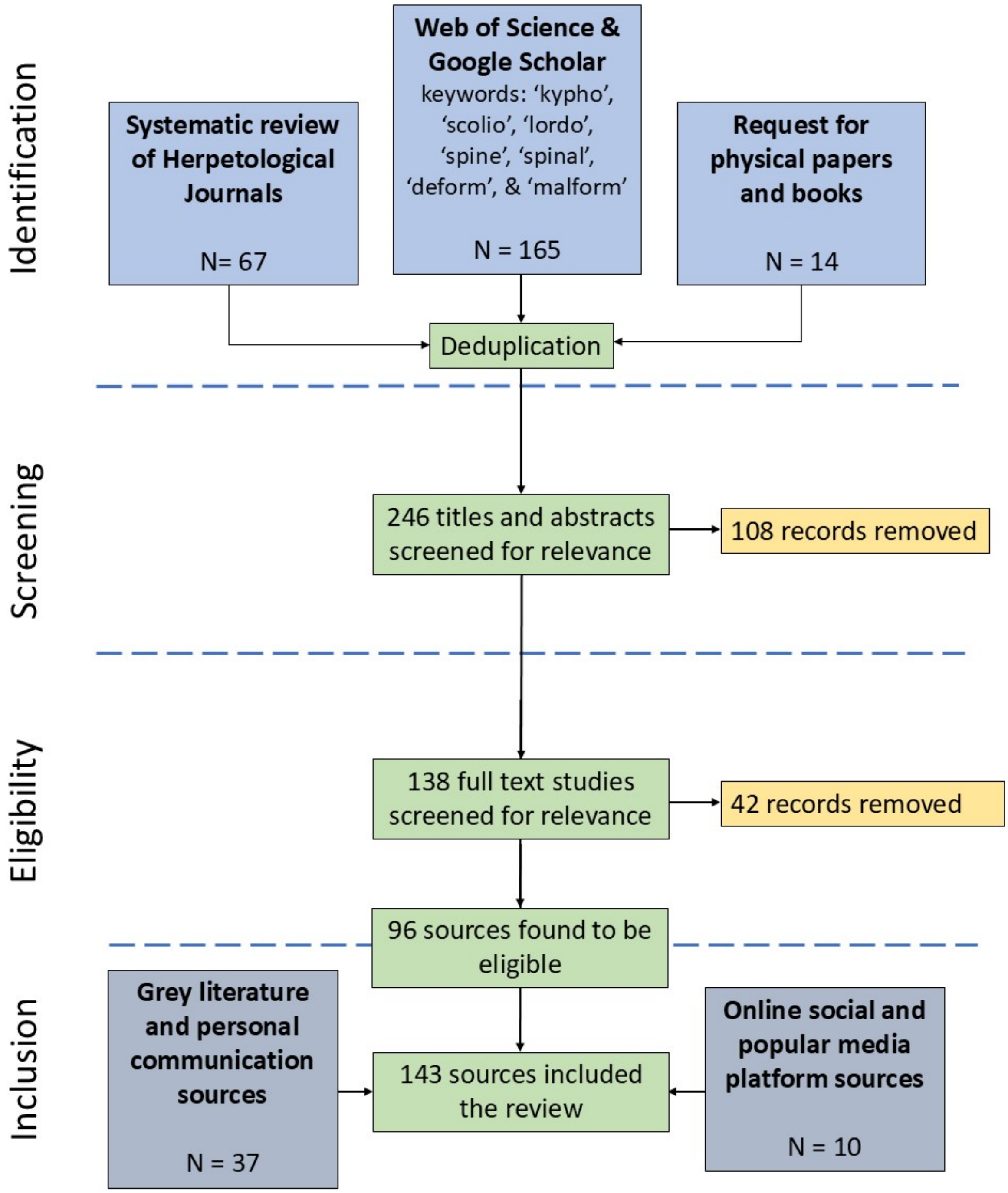

Supplementary Figure S1. PRISMA diagram summarizing the literature search.

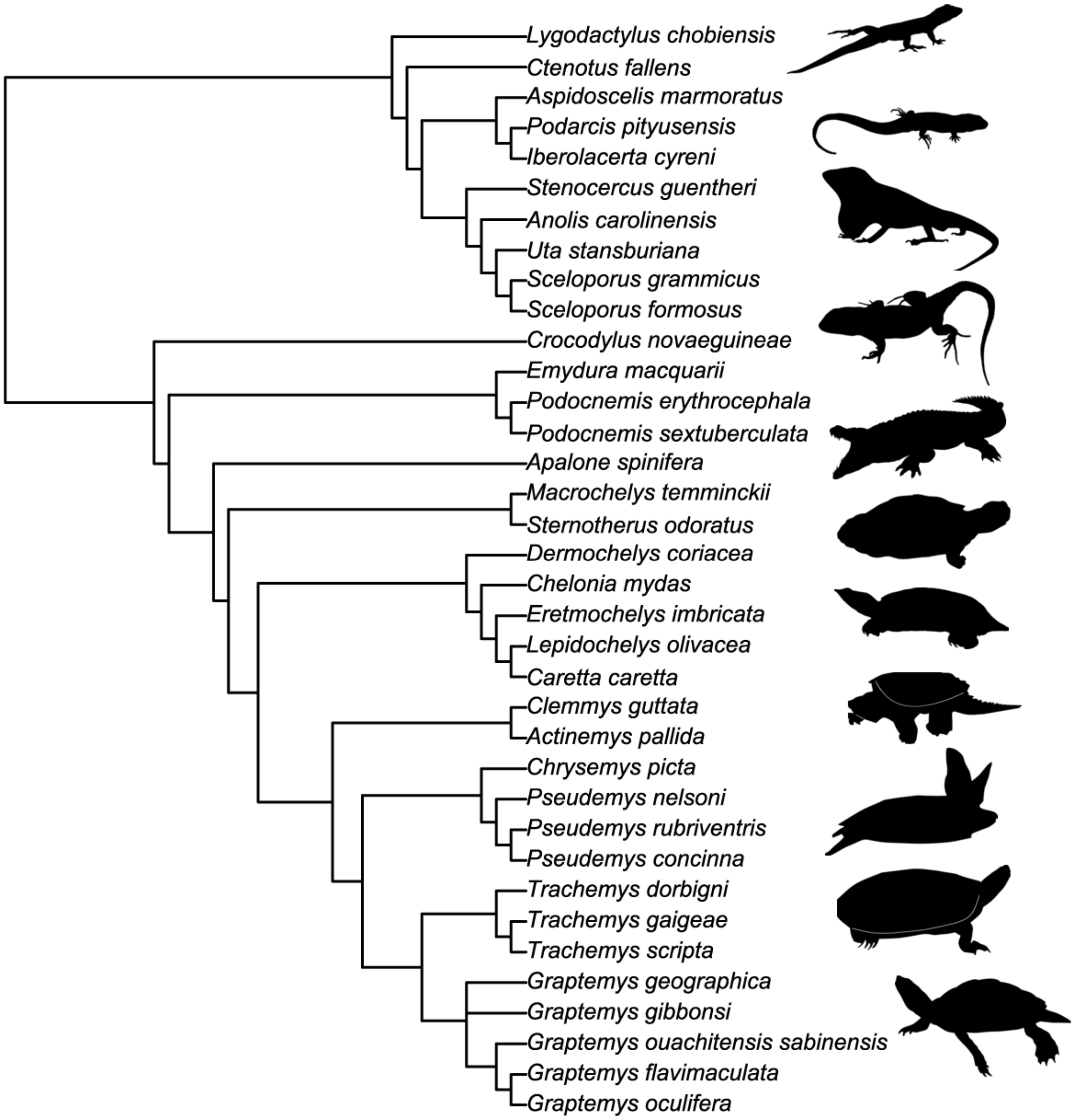

Supplementary Figure S2. Graphical summary of phylogenetic distribution of 35 species included in the meta-analyses. Note that, for compatibility with the Open Tree of Life database, the Suwannee snapping turtle (*Macrochelys suwanniensis*) was treated as a synonym of the alligator snapping turtle (*M. temminckii*). The tree was created using the 'rotl' package [92] in R. Silhouettes depicting selected taxa obtained from phylopic.org.

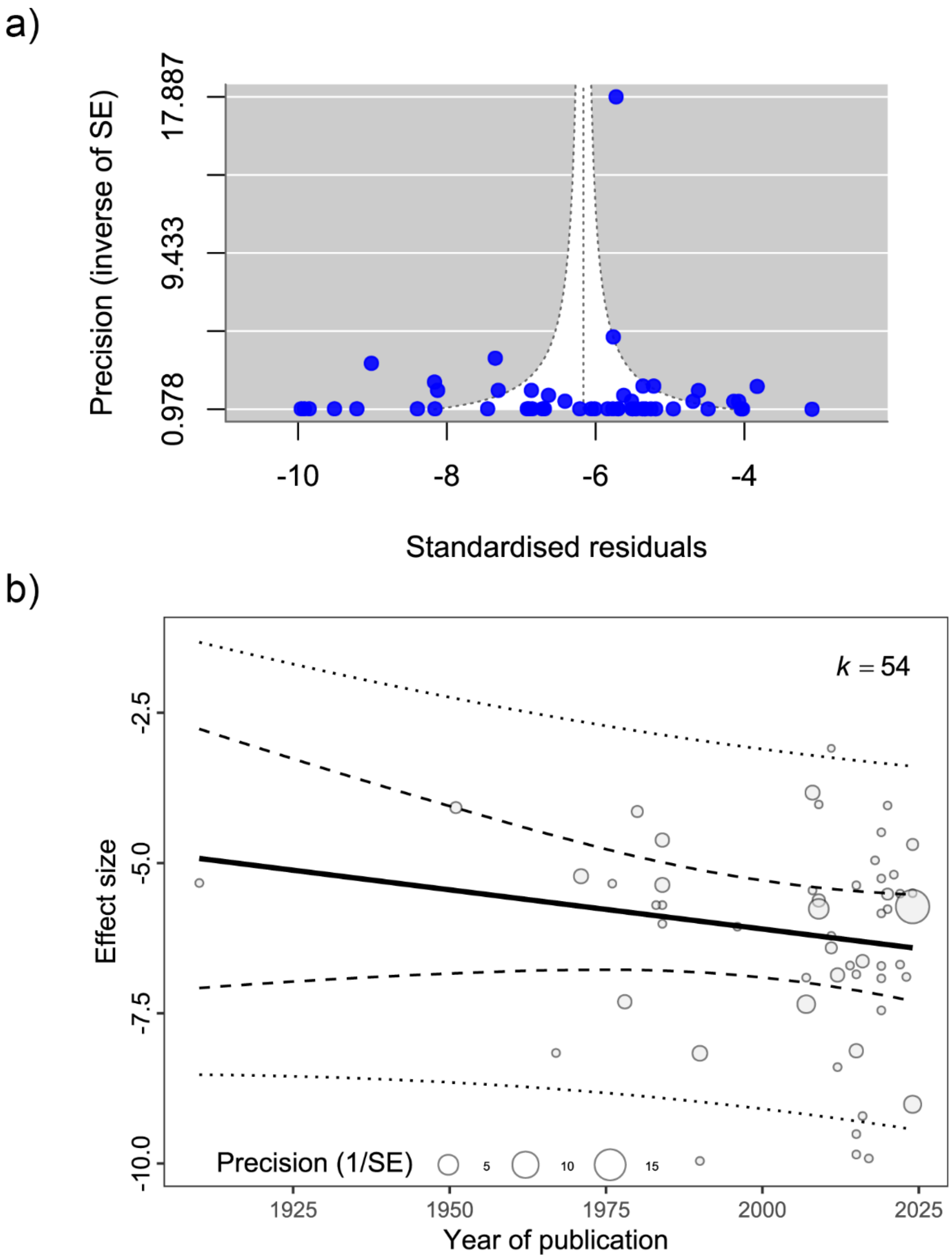

Supplementary Figure. S3. Funnel plot and relationships between effect sizes and publication year. a) Funnel plot using effect size and its inverse standard error; b) the relationship between effect sizes and publication year. In b) circle sizes are scaled accordingly to precision, and k represents the number of effect sizes. Fitted regression line is shown as a straight line, and 95% confidence and prediction intervals are shown as dashed and dotted lines, respectively.

Supplementary Table S2. Occurrence and prevalence of spinal deformations in the currently known families of reptiles. Families are consistent with the most recent classifications from The Reptile Database (Uetz *et al.* [83], accessed 22 May 2025). Numbers in parentheses are the number of species observed from the literature search to have spinal deformities and the number of known species in the family.

Order	Family	Species	Abnormality	Prevalence (%)	Reference
**Crocodilia**	Crocodylidae (2/17)	*Crocodylus acutus*	scoliosis		Rossmann [108]
		Crocodylus novaeguineae	kyphosis	0.47	Montague [194]
	Gavialidae (0/2)	-	-	-	-
	Alligatoridae (1/8)	*Alligator mississippiensis*	kyphosciliosis	-	Elsey & Stelly [36]
**Rhynchocephalia**	Sphenodontidae (0/1)	-	-	-	-
**Squamata**	Agamidae (7/602)	*Agama anchietae*	scoliosis	-	Grogan [77]
		Agama bibronii	scoliosis	-	Ahboucha & Gamrani[82]
		Calotes goetzi	kyphoscoliosis	-	Henrik Bringsøe; *pers. comm.*
		Calotes versicolor	scoliosis	-	Henrik Bringsøe; *pers. comm.*
		Calotes versicolor	kyphosis	-	Datta & Hasan [139]
		Diploderma chapaense	scoliosis	-	Norval *et al.* [150]
		Trapelus mutabilis	kyphoscoliosis	-	Böhme [161]
	Chamaeleonidae (1/234)	*Furcifer pardalis*	kyphoscoliosis	-	Gehring [181]
	Corytophanidae (0/11)	-	-	-	-
	Crotaphytidae (0/12)	-	-	-	-
	Anolidae (4/435)	*Anolis carolinensis*	scoliosis	0.16	Stuart, *online, pers. comm.*
		Anolis cybotes	kyphoscoliosis	-	Tiburcio *et al.* [214]
		Anolis sagrei	scoliosis	-	Campbell [165)
		Anolis sagrei	scoliosis	-	Losos, *online*
		Anolis sericeus	kyphoscoliosis	-	Orós *et al.* [199]
	Hoplocercidae (0/22)	-	-	-	-
	Iguanidae (2/45)	*Sauromalus hispidus*	kyphosis	-	Hirst *et al.* [147]
		Cyclura cychlura	kyphoscoliosis	-	Owens & Knapp [156]
	Leiocephalidae (0/30)	-	-	-	-
	Leiosauridae (0/36)	-	-	-	-

Liolaemidae (6/342)	*Liolaemus baguali*	kyphosis	-	Feltrin *et al.* [23]
	Liolaemus darwinii	kyphoscoliosis	-	Avellá Machado & Acosta [146]
	Liolaemus fittkaui	kyphoscoliosis	-	Octavio Jiménez-Robles; pers. comm.
	Liolaemus huacahuascianus	kyphosis	-	Halloy & Laurent [186]
	Liolaemus koslowskyi	kyphoscoliosis	-	Avila *et al.* [68]
	Liolaemus petrophilus	scoliosis	-	Frutos *et al.* [79]
Opluridae (0/8)	-	-	-	-
Phrynosomatidae (9/170)	*Phrynosoma douglasii*	kyphosis?	-	Jackson Shedd; pers. comm.
	Sceloporus formosus	kyphoscoliosis	1.72	Castillo-Juárez *et al.* [167]
	Sceloporus graciosus	kyphoscoliosis	-	Valdez-Villavicencio *et al.* [70]
	Sceloporus grammicus	kyphoscoliosis	0.41	Hernández *et al.* [145]
	Sceloporus magister	kyphoscoliosis	-	Dennis Caldwell; *pers. comm.*
	Sceloporus marmoratus	kyphoscoliosis	-	Chávez-Cisneros *et al.* [169]
	Sceloporus torquatus	kyphosis	-	Pérez-Delgadillo *et al.* [69]
	Sceloporus undulatus	kyphoscoliosis	-	Mitchell & Georgel [135]
	Uta stansburiana	scoliosis	-	Stacey Schenkel; pers. comm.
	Uta stansburiana	scoliosis	0.03	Tinkle [215]
Polychrotidae (0/8)	-	-	-	-
Tropiduridae (2/149)	*Plica umbra*	kyphoscoliosis	-	Carvalho *et al.* [128]
	Stenocercus guentheri	kyphoscoliosis	0.69	Ramírez-Jaramillo [74]
Gekkonidae (4/1680)	*Lepidodactylus lugubris*	scoliosis		Chan *et al.* [168]
	Lygodactylus chobiensis	scoliosis	0.33	Simbotwe [78]
	Phelsuma grandis	rhoecosis	-	Caceres *et al.* [163]
	Phelsuma modesta	kyphoscoliosis	-	Gehring *et al.* [182]
Carphodactylidae (0/34)	-	-	-	-
Diplodactylidae (0/202)	-	-	-	-
Eublepharidae (0/48)	-	-	-	-
Phyllodactylidae (0/168)	-	-	-	-
Sphaerodactylidae (0/238)	-	-	-	-
Pygopodidae (0/47)	-	-	-	-

Cordylidae (0/69)	-	-	-	-
Gerrhosauridae (0/39)	-	-	-	-
Scincidae (5/1785)	*Ctenotus fallens*	scoliosis	0.12	Bateman *et al.* 81
	Lampropholis guichenoti	scoliosis	-	Philip Bateman; *pers. comm.*
	Marisora brachypoda	kyphosis	-	Arrivillaga & Brown [157]
	Sphenomorphus melanopogon	scoliosis		Kaiser *et al.* [189]
	Trachylepis seychellensis	kyphosis	-	Petrişor Mădălina; *pers. comm.*
Xantusiidae (0/38)	-	-	-	-
Alopoglossidae (0/32)	-	-	-	-
Gymnophthalmidae (0/297)	-	-	-	-
Lacertidae (5/387)	*Iberolacerta cyreni*	kyphosis	0.55	Horváth *et al.* [71]
	Lacerta agilis	kyphoscoliosis	-	Geus [183]
	Lacerta agilis	scoliosis	-	Gordeev *et al.* [185]
	Podarcis pityusensis	kyphosis	0.2	Garin-Barrio *et al.* [67]
	Psammodromus hispanicus	scoliosis?	-	Doru Panaitescu; *pers. comm.*
	Timon Lepidus	kyphosis	-	Berdún & Bisbal-Chinesta [159]
	Timon lepidus	kyphosis	-	Martínez-Rodríguez; *online*
Teiidae (2/173)	*Aspidoscelis tigris*	scoliosis	-	Heyborne [187]
	Aspidoscelis marmoratus	scoliosis	0.1	Rocha *et al.* [208]
Anguidae (0/88)	-	-	-	-
Diploglossidae (0/90)	-	-	-	-
Xenosauridae (0/14)	-	-	-	-
Amphisbaenidae (0/184)	-	-	-	-
Bipedidae (0/3)	-	-	-	-
Blanidae (0/7)	-	-	-	-
Cadeidae (0/2)	-	-	-	-
Rhineuridae (0/1)	-	-	-	-
Trogonophidae (0/6)	-	-	-	-
Helodermatidae (0/5)	-	-	-	-

Lanthanotidae (0/1)	-	-	-	-
Varanidae (0/88)	-	-	-	-
Shinisauridae (0/1)	-	-	-	-
Dibamidae (0/28)	-	-	-	-
Acrochordidae (0/3)	-	-	-	-
Anomochilidae (0/3)	-	-	-	-
Cylindrophiidae (0/15)	-	-	-	-
Uropeltidae (0/73)	-	-	-	-
Loxocemidae (0/1)	-	-	-	-
Pythonidae (0/38)	-	-	-	-
Xenopeltidae (0/3)	-	-	-	-
Boidae (1/67)	*Sanzinia madagascariensis*	kyphoscoliosis, scoliosis	-	Progscha & Lehmann [130]
Colubridae (7/2156)	*Arizona elegans*	kyphoscoliosis	-	Alvarez *et al.* [140]
	Coronella austriaca	lordosis	-	Najbar *et al.* [118]
	Coronella austriaca	scoliosis?		Golder [184]
	Nerodia sipedon	scoliosis?	-	Wallach & Salmon [131]
	Oxybelis microphthalmus	scoliosis	-	Nieto-Toscano & Martínez-Coronel [197]
	Pantherophis gloydi	kyphosis	-	Brazeau *et al.* [138]
	Thamnopis radix	rhoecosis	-	Smith & Fitzgerald [9]
	Thamnopis sirtalis	scoliosis	-	Gray *et al.* [52]
Atractaspididae (0/68)	-	-	-	-
Cyclocoridae (0/8)	-	-	-	-
Micrelapidae (0/6)	-	-	-	-
Lamprophiidae (0/93)	-	-	-	-
Prosymnidae (0/19)	-	-	-	-
Psammodynastidae (0/2)	-	-	-	-
Psammophiidae (0/57)	-	-	-	-
Pseudaspididae (0/2)	-	-	-	-
Pseudoxyrhophiidae (0/89)	-	-	-	-

	Elapidae (0/416)	-	-	-	-
	Anomalepididae (0/23)	-	-	-	-
	Gerrhopilidae (0/29)	-	-	-	-
	Typhlopidae (0/282)	-	-	-	-
	Leptotyphlopidae (0/146)	-	-	-	-
	Xenotyphlopidae (0/1)	-	-	-	-
	Aniliidae (0/1)	-	-	-	-
	Bolyeriidae (0/2)	-	-	-	-
	Homalopsidae (0/60)	-	-	-	-
	Pareidae (0/45)	-	-	-	-
	Tropidophiidae (0/37)	-	-	-	-
	Viperidae (6/405)	*Bothrops ammodytoides*	kyphoscoliosis		Feldman et al. [177]
		Bothrops jararaca	kypholordosis, kyphoscoliosis, kyphosis, lordoscoliosis, lordosis, rhoecosis, scoliosis	-	Carvalho [35]
		Crotalus durissus	kyphoscoliosis, kyphosis, lordosis, rhoecosis, scoliosis	-	Carvalho [35]
		Vipera ammodytes	scoliosis	-	Ivo Peranic; *pers. comm.*
		Vipera graeca	scoliosis?	-	Bálint Üveges; *pers. comm.*
		Vipera ursinii moldavica	scoliosis?		Bálint Üveges; *pers. comm.*
		Vipera ursinii rakosiensis	scoliosis? kyphosis?		Bálint Üveges; *pers. comm.*
	Xenodermidae (0/37)	-	-	-	-
	Xenophidiidae (0/2)	-	-	-	-
**Testudines**	Emydidae (27/58)	*Actinemys pallida*	kyphosis	0.91	Valdez-Villavicencio *et al.* [141]

Chrysemys picta	kyphosis	0.54	Ernst [175]
Chrysemys picta	kyphosis	-	Pavalko [202]
Chrysemys picta	kyphosis	-	Thomas Spence; *online*
Chrysemys picta bellii	scoliosis	1.56	Macculloch [191]
Chrysemys picta bellii	kyphosis	0.23	Stuart [135]
Chrysemys picta bellii × *marginata*	kyphosis	-	Necker [17]
Chrysemys picta marginata	kyphosis	-	Ernst [176]
Chrysemys picta marginata	kyphosis	0.11	Moldowan *et al.* [132]
Chrysemys picta marginata	kyphosis	-	Werner Jr. [221]
Chrysemys picta picta	lordosis	0.12	Mitchell [193]
Trachemys yaquia	kyphosis	-	Plymale *et al.* [205]
Clemmys guttata	kyphosis	0.48	Ernst [176]
Glyptemys insculpta	kyphosis	-	Harding & Bloomer [143]
Glyptemys insculpta	scoliosis	-	Jonathan Mays; *online*
Deirochelys reticularia chrysea	kyphoscoliosis	-	Mitchell & Johnston [72]
Emys orbicularis	kyphosis?	-	Rita Babos; *online, pers. comm.*
Emys orbicularis	lordosis	-	Valdeón *et al.* [218]
Graptemys flavimaculata	kyphosis	0.09	Mitchell *et al.* [92]
Graptemys flavimaculata	scoliosis	-	Selman [210]
Graptemys geographica	kyphoscoliosis	-	Bennett & Litzgus[158]
Graptemys geographica	kyphosis	1.11	Mitchell *et al.* [92]
Graptemys geographica	kyphosis	0.06	Mitchell *et al.* [92]
Graptemys geographica	kyphosis	-	Mitchell *et al.* [92]
Graptemys geographica	kyphosis	0.12	Mitchell *et al.* [92]
Graptemys gibbonsi	kyphosis	0.29	Mitchell *et al.* [92]
Graptemys oculifera	kyphosis	0.1	Harding & Bloomer [143]
Graptemys ouachitensis	kyphosis	-	Carpenter [133]

	Graptemys sabinensis	kyphosis	0.46	Loque Jr. *et al.* [190]
	Graptemys sabinensis	kyphoscoliosis	0.46	Loque Jr. *et al.* [190]
	Graptemys versa	kyphosis	-	Franklin [179]
	Malaclemys terrapin centrata	lordosis?, scoliosis?	-	Hildebrand [81]
	Malaclemys terrapin centrata	kyphosis, lordosis	-	Selman [211]
	Podocnemis erythrocephala	kyphosis	0.02	Bernhard *et al.* [137]
	Pseudemys concinna suwanniensis	kyphoscoliosis	0.13	Mitchell & Johnston [193]
	Pseudemys gorzugi	kyphosis	-	Waldon [219]
	Pseudemys gorzugi	kyphoscoliosis?	-	Zymonas [225]
	Pseudemys nelsoni	kyphoscoliosis	0.31	Jackson & Zappalorti [93]
	Pseudemys peninsularis	kyphosis	-	Donini *et al.* [171]
	Pseudemys peninsularis	kyphosis	-	Mark Witwer; *online*
	Pseudemys rubriventris	kyphosis	0.4	Jackson & Zappalorti [93]
	Terrapene carolina	scoliosis	-	Palis [201]
	Terrapene ornata	kyphoscoliosis	-	Fox [178]
	Terrapene triunguis	kyphosis	-	Black [160]
	Trachemys dorbigni	kyphosis	1.75	Bujes [162]
	Trachemys gaigeae gaigeae	kyphoscoliosis?	0.43	Stuart & Painter [212]
	Trachemys gaigeae gaigeae	kyphosis	2.13	Stuart & Painter [212]
	Trachemys scripta elegans	kyphoscoliosis	-	Elsey *et al.* [142]
	Trachemys scripta elegans	kyphosis	0.06	Tucker *et al.* [76]
	Trachemys scripta elegans	kyphosis	-	Carr [166]
	Trachemys scripta elegans	kyphoscoliosis	-	Enge [174]
Testudinidae (0/47)				
Geoemydidae (0/75)				

Platysternidae (0/1)				
Carettochelydae (0/1)				
Trionychidae (7/36)	*Apalone ferox*	kyphosis	-	Nixon & Smith [198]
	Apalone ferox	kyphosis	-	Pritchard [207]
	Apalone ferox	kyphosis	-	Taylor & Mendyk [213]
	Apalone mutica	kyphosis	-	Smith [24]
	Apalone mutica mutica	kyphosis	-	Webb [220]
	Apalone spinifera	kyphosis	-	Burke [155]
	Apalone spinifera	kyphosis	-	Cahn [164]
	Apalone spinifera	kyphosis	-	Smith [24]
	Apalone spinifera	kyphosis	-	Webb [220]
	Apalone spinifera emoryi	kyphosis	-	Smith [24]
	Apalone spinifera emoryi	kyphosis	-	Stuart [134]
	Apalone spinifera emoryi	kyphosis	-	Webb [220]
	Apalone spinifera hartwegi	kyphosis	-	Webb [220]
	Apalone spinifera spinifera	kyphosis	1.67	Neill [196]
	Apalone spinifera spinifera	kyphosis	-	Webb [220]
	Apalone spinifera spinifera	kyphosis	-	White & Murphy [222]
	Lissemys punctata punctata	kyphosis	-	Duda & Gupta [172]
	Palea steindachneri	kyphosis	-	Gressit [15]
	Pelodiscus sinensis	kyphosis	-	Vogt [12]
	Trionyx triunguis	kyphosis	-	Mertens [16]
	Trionyx triunguis	lordosis	-	Pritchard [207]
Chelydridae (3/5)	*Chelydra serpentina*	kyphosis, scoliosis	-	Bell [34]
	Chelydra serpentina	kyphosis	-	Cahn [164]

	Chelydra serpentina	kyphoscoliosis	-	Schachner *et al.* [37]
	Chelydra serpentina	kyphosis	-	Wilhoft [223]
	Macrochelys suwanniensis	kyphosis	0.4	Enge *et al.* [173]
	Macrochelys suwanniensis	kyphosis	-	Jake Scott; *online*
	Macrochelys temminckii	kyphosis	0.52	Pearson *et al.* [203]
	Macrochelys temminckii	kyphosis	-	Pritchard [206]
Dermatemydidae (0/1)				
Kinosternidae (1/33)	*Sternotherus odoratus*	kyphosis	0.1	Iverson [188]
	Sternotherus odoratus	kyphosis	-	Nixon & Smith [198]
	Sternotherus odoratus	kyphosis	-	Saumure [209]
Cheloniidae (4/6)	*Caretta caretta*	scoliosis	0.48	Coker [170]
	Caretta caretta	kyphosis	0.005	Drennen [129]
	Caretta caretta	scoliosis	0.03	Drennen [129]
	Caretta caretta	kyphosis, kyphosis?, lordosis	-	Inwater Research Group Inc.; *pers. comm.*
	Caretta caretta	scoliosis	-	Nardini *et al.* [195]
	Caretta caretta	kyphosis	-	Orós *et al.* [199]
	Caretta caretta	kyphoscoliosis?	-	Wilson *et al.* [224]
	Chelonia mydas	kyphosis	-	Inwater Research Group Inc.; *pers. comm.*
	Chelonia mydas	kyphosis	-	Moosnipol Educación Ambiental y Conservación; *online*
	Chelonia mydas	kyphosis	0.98	Rhodin *et al.* [91]
	Chelonia mydas	lordosis	0.25	Rhodin *et al.* [91]
	Chelonia mydas	kyphosis	0.33	Sönmez & Sağol [109]
	Chelonia mydas	scoliosis	0.01	Sönmez & Sağol [109]
	Eretmochelys imbricata	kyphosis	0.005	Bárcenas-Ibarra *et al.* [32]
	Eretmochelys imbricata	kyphosis	-	Inwater Research Group Inc.; *pers. comm*

	Lepidochelys olivacea	kyphosis	0.31	Bárcenas-Ibarra & Maldonado-Gasca [31]
	Lepidochelys olivacea	kyphosis	0.007	Bárcenas-Ibarra *et al.* [32]
	Lepidochelys olivacea	scoliosis	0.03	Bárcenas-Ibarra *et al.* [32]
	Lepidochelys olivacea	scoliosis	0.005	Bárcenas-Ibarra *et al.* [33]
	Lepidochelys olivacea	kyphosis	0.33	Rhodin *et al.* [91]
Dermochelyidae (1/1)	*Dermochelys coriacea*	kyphosis	0.07	Fretey [180]
	Dermochelys coriacea	kyphosis	4.34	Honarvar *et al.* [136]
Chelidae (2/67)	*Elseya irwini*	kyphoscoliosis	-	Turner [217]
	Emydura macquarii krefftii	kyphosis	0.36	Trembath [216]
Pelomedusidae (0/27)	-	-	-	-
Podocnemididae (1/8)	*Podocnemis sextuberculata*	kyphosis	0.01	Perrone [204]
